# Optical Band Gap Tuning, DFT Understandings, and Photocatalysis Performance of ZnO Nanoparticle-Doped Fe Compounds

**DOI:** 10.3390/ma16072676

**Published:** 2023-03-28

**Authors:** Gharam A. Alharshan, Abdelaziz Mohamed Aboraia, Mohamed A. M. Uosif, Ibrahim M. Sharaf, Essam R. Shaaban, Mohamed Saad, Hussain ALMohiy, Mohamed M. Elsenety

**Affiliations:** 1 Physics Department, College of Science, Princess Nourah Bint Abdulrahman University, P.O. Box 84428, Riyadh 11671, Saudi Arabia; 2 Physics Department, Faculty of Science, Al-Azhar University, Assiut 71542, Egypt; 3 Energy Storage Research Laboratory (ESRL), Physics Department, Faculty of Science, Al-Azhar University, Assiut 71542, Egypt; 4 Physics Department, College of Science, Jouf University, Sakaka 72388, Saudi Arabia; 5 Department of Radiological Science, Faculty of Applied Medical Science, King Khalid University, Abha P.O. Box 9004, Saudi Arabia; 6 Department of Physics, Faculty of Science, Mansoura University, Mansoura 35516, Egypt; 7 Department of Chemistry, Faculty of Science, Al-Azhar University, Cairo 11884, Egypt

**Keywords:** zinc oxide, nanoparticles, doped materials, photocatalysis, water purification

## Abstract

Iron-doped Zinc oxide nanoparticles were produced by the sol-gel combustion method. This study aims to see how iron doping affects the structural, optical, and photocatalytic characteristics of ZnO composites. XRD examined all samples to detect the structural properties and proved that all active materials are a single hexagonal phase. The morphology and particle size were investigated by TEM. Computational Density functional theory (DFT) calculation of the band structure, density of state, and charge distributions for ZnO were investigated in comparison with ZnO dope iron. We reported the application results of ZnO doped Fe for Methylene blue dye removal under photocatalytic degradation effect. The iron concentrations affect the active material’s band gap, producing higher photocatalytic performance. The acquired results could be employed to enhance the photocatalytic properties of ZnO.

## 1. Introduction

Water contamination should receive increasing attention as an environmental concern as the world’s industrialization accelerates [1,2]. Dye sewages have a significant detrimental impact on the ecosystem owing to their toxicity and non-biodegradability [3,4]. Methylene blue (M.B.) is a dye that can be used in textile dyeing [5,6]. It is vital for a green environment to develop ways and technologies to eliminate this industrial effluent [7]. Recently, semiconductors, for instance: cadmium sulfide, titanium dioxide, copper oxide, vanadium oxide, tungsten oxide, and zinc oxide, have been employed to photo-degrade different odyes [8,9,10]. Among the semiconductors listed above, ZnO is considered a significant photocatalyst for modern, efficient oxidation techniques due to its abundant element, cheap, affordable, and adaptable n-type material [11]. Despite its broad bandgap, ZnO can absorb a bigger proportion of the U.V. spectrum area and has a stronger photocatalytic capacity compared to TiO_2_ in the breakdown of dyes.

Zinc oxide materials are an adaptable II-VI group and have a direct bandgap of ~3.4 eV and excellent excitonic binding energy at room temperature [12,13]. Due to their unique optical, electronic, electrical, and chemical properties required for developing nanoscale and optoelectronic devices as chemical sensors, ZnO nanocrystals have gained attention recently [14,15]. 

In addition, zinc oxide is a semiconductor material that has a number of interesting properties that make it attractive for a variety of applications. One of these properties is its high quantum efficiency, which refers to the ability of a material to convert absorbed photons (particles of light) into electrons. This is important for applications such as solar cells, which aim to convert light into electricity [16,17] efficiently. ZnO also has good photo-corrosion and photochemical characteristics, which means that it is resistant to degradation when exposed to light, and can be used to trigger chemical reactions when irradiated with light. This makes it a good candidate for use in photocatalysis.

Optical band gap tuning refers to the process of altering the energy required for electrons to be excited from the valence band to the conduction in a semiconductor material. This can be done by introducing impurities (also known as dopants) into the semiconductor, which can alter its electronic properties. However, tuning the energy gap of ZnO semiconductor materials has piqued the interest of researchers in recent years due to improvements in their characteristics as well as advancements in device performance [18,19]. This advancement is capable of fine-tuning its band gap by doping with a range of transition metals (T.M.) such as Co, Cu, Ni, Hf, Cd, Ag, and Fe [20,21]. 

ZnO nanoparticles have been of interest for their potential use in photocatalysis. By doping these particles with Fe compounds, it is possible to tune the electronic properties of the ZnO nanoparticles, which can affect their performance as photocatalysts. We were inspired by the contradictory findings to investigate ferromagnetic Fe-doped ZnO nanoparticles and explain their physical and structural features. Details in the nanostructure, for instance: crystal structure, particle shape, size, and flaws, are widely recognized to be crucial for many applications. Using the sol-gel combustion process, we created a series of Fe-doped ZnO nanoparticles with x = 0, 0.03, 0.05, 0.07, 0.1 and 0.15 mol percent of Fe. The iron substitution was examined for its implications on structural, morphological, and optical characteristics, thus the photocatalytic activity. All the microstructures of the samples were examined using X-ray diffraction (XRD), and transmission electron microscopy. The optical band gap was investigated by U.V.–Vis spectroscopy.

## 2. Experimental Technique

### 2.1. Synthesis

The sol-gel combustion route has synthesized pristine ZnO and ZnO nanoparticles doped with different concentrations of Fe. The pure ZnO was prepared by dissolving 1 mal of Zn(NO_3_)_2_·6H_2_O in a suitable volume of DMF with 2 mal citric acid as a combustion fuel in double distilled water at a fixed ratio of 1:2. The yield was stirred at 80 °C. The powder was placed in a muffle furnace for two hours at 600 °C. To prepare the rest samples doping with different concentrations of Fe, the same protocol of undoped ZnO was fellow for doping with varying ratios of molar Fe(NO_3_)_3_·9H_2_O. A brown-to-black, voluminous and fluffy product was easily ground to a powder.

### 2.2. Characterization Methods

X-ray powder diffraction was obtained using a Philips X-ray diffractometer model PW 1710 with CuKα radiation (λ = 1.5405 Å), an operational applied voltage of 40 kV, and a current of 30 mA. The scanning rate was kept constant at 0.02° per minute in the 4–80 range. The KBr pellet technique was used to record FTIR spectra for varied compositions of Fe-doped ZnO nanoparticles using a NICOLET FTIR 6700 spectrometer in the wavenumber range 400–4000 cm^−1^. The optical proportions of the mixture were determined using a twin-beam JASCO 670 UV-Visible Spectrophotometer between 200 and 1000 nm. 

### 2.3. The Photocatalytic Performance of All Active Materials

Under U.V. irradiation, photocatalytic investigations of ZnO were evaluated for M.B. degradation. ZnO photocatalyst and M.B. solution were mixed for 100 min in a dark box before radiation exposure in order to achieve an adsorption/desorption equilibrium between them. ZnO adsorbed with M.B. molecules results in a straightforward M.B. reaction with electron and hole pairs. Furthermore, the experiments were carried out using a hero lab UV-15 S/L at ambient temperature. A closed dark reactor was used, and the distance between UV-lamp and M.B.- ZnO beaker was 10 cm. M.B. photodegradation was also tested by measuring the absorbance of the M.B. solution at 665 nm. The following equation can be used to calculate the effectiveness of photodegradation or removal [22]:

(1) $$Removal\;\%=\frac{\left(C_{i}-C_{f}\right)}{C_{i}}\times 100\;$$

After the photocatalytic degradation process has been completed, the starting and final M.B. concentrations (*C_i_* and *C_f_*) are determined. According to a standard experimental protocol, 1 milliliter of MB-dye (10 ppm) solution was combined with 99 milliliters of deionized water to make up a Pyrex beaker containing 10 mg of ZnO. A regular period was established for filtering the material, which was then centrifuged at 5000 RPM for 10 min before being evaluated using UV-Vis spectroscopy when it was completed. A series of photocatalytic studies were conducted to evaluate the effects of pH, contact duration, dosage, and temperature on photocatalytic activity. The consumed ZnO was also collected and mixed with 0.1 M HNO_3_ before being washed with deionized water under ultrasonic conditions twice to remove any leftover M.B. after the photocatalytic process was completed. The photocatalyst ZnO that has been regenerated can be utilized up to six times with excellent efficiency.

## 3. Results and Discussion

### 3.1. X-ray Diffraction Analysis

**Figure 1:**
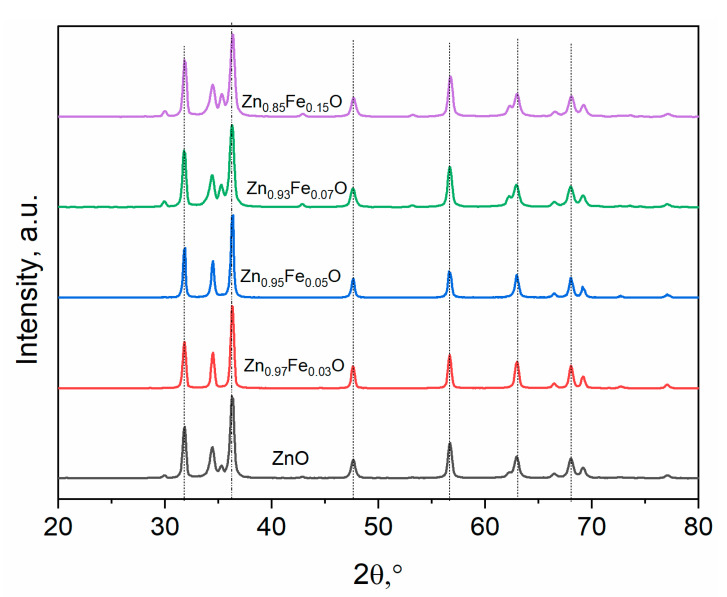
XRD patterns of Zn_1−x_Fe_x_O.

Figure 1 show powder X-ray diffraction of undoped and Fe-doped ZnO nanostructures. Zn_1−x_Fe_x_O (x = 0, 0.03, 0.05, 0.07, 0.1, and 0.15) samples exhibit pure hexagonal structure of ZnO and space group *P*6_3_*mc*, (PDF # 00-900-4178). There was no contamination, i.e., Fe cluster or Fe_2_O_3_, suggesting that the iron content is below the solubility limitations. However, it is possible that secondary amorphous phase clusters or precipitates could form that are too tiny to be detected by XRD measurements. As shown in Figure 1, the diffraction peak of 7% Fe-doped ZnO at 2Ɵ = 34.305°) has migrated towards lower angles as compared to pure ZnO (2Ɵ = 34.355°). This is because the ionic radii of Fe are larger than that of Zn. Moreover, the peak shifting may refer to the crystallography of the Zn position that has been occupied by Fe ions in the host lattice of ZnO and strain improved in the lattice. However, a 15% or higher increase in the amount of Fe caused the formation of a new phase from Fe_3_O_4_. 

**Figure 2:**
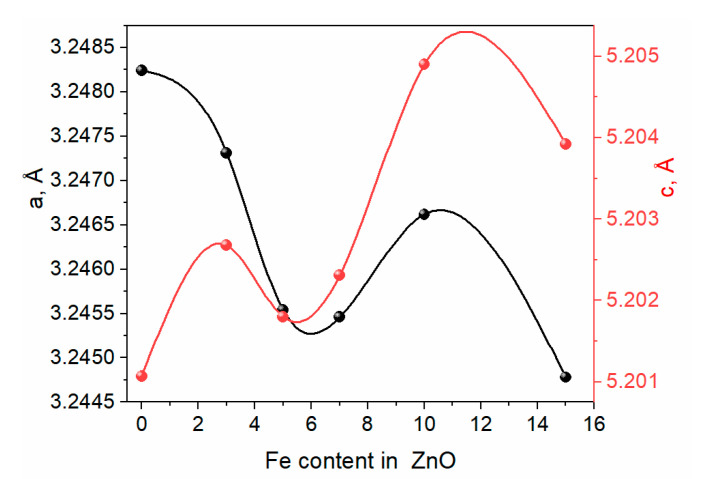
Variation of lattice parameters drives from Rietveld refinements (XRD patterns of Zn1-xFexO (x = 0 (a, 0.03, 0.05, 0.07, and 0.15).

The Rietveld refinement approach has been used to better understand how Fe ions affect the lattice properties of undoped ZnO. Rietveld-refined X-Ray Diffraction patterns of the pure ZnO and Fe-doped ZnO are exhibited in Figure 2. The pseudo-Voigt function was used to illustrate the line profiles of XRD. Observed and calculated values matched well, as can be recognized from the figures, and are consistent with previous studies.

Further, the lattice constants (*a*, *c*), unit cell volume (*V*), degree of distortion (*R*), and bond length (along the *c*-axis) were carefully calculated using the Rietveld analysis provided in the package. The obtained data are plotted in Figure 2. 

One may suppose the expansion of lattice parameters *c* and shrinking *a* as a result of peak shifting in the direction of lower 2*θ* angles.

$$c=\frac{\lambda }{sin\theta }\;,\;a=\frac{\lambda }{3\;sin\theta }$$

In the refined XRD patterns, the lattice parameters “*a*” decrease with the increase in the amount of Fe. In contrast, “*c*” lattice parameters increase with the increase in the amount of Fe. This may be ascribed to various factors, like the concentration of dopant, external strains improved owing to temperature, defects (vacancies, dislocation, interstitial), and the difference among the ionic radii of Fe and Zn ions.

### 3.2. TEM Photos

**Figure 3:**
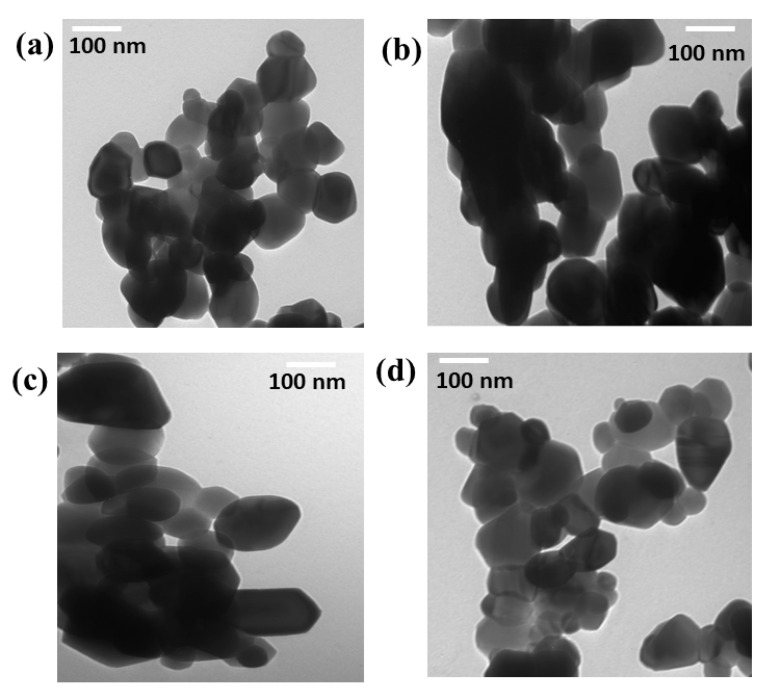
TEM photos of (**a**) pure ZnO, (**b**) 3% Fe doped ZnO, (**c**) 5% Fe doped ZnO, (**d**) 15% Fe doped ZnO.

According to Figure 3a–d, TEM examinations were undertaken to determine the size and microstructure of the pristine ZnO N.P.s as well as the ZnO doped with various Fe concentrations. According to Figure 3a, the average size is less than 50 nm. However, the average size of the materials was increased by an increase in the amount of Fe. The TEM photos exhibited a single phase of ZnO doped with different ratios of Fe and revealed no additional phases from Fe; thus, the iron has been solved in the ZnO very well.

### 3.3. Optical Properties

**Figure 4:**
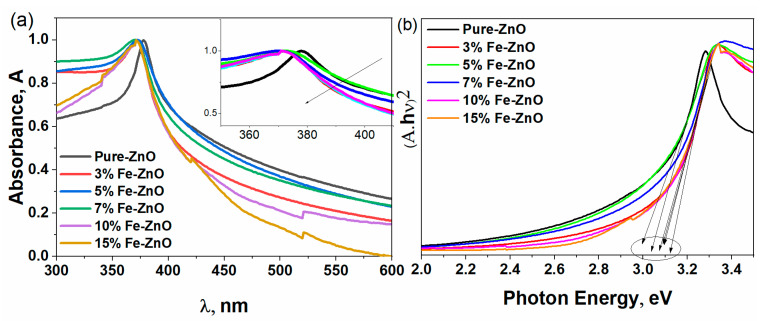
(UV-vis) absorption spectra of Zn_1−x_Fe_x_O, the absorption band gets a blue shift (370–380 nm), as shown in (**a**), (**b**) Tauc’s relation.

Figure 4a,b displays the absorption and optical band energy gap of Zn_1−x_Fe_x_O nanocrystals. The absorption peak of pristine ZnO was 380 nm in the region Uv, and the doping of Fe concentrations significantly decreased the absorption band to 370 nm. The absorption band gets a blue shift (370–380 nm), as shown in Subfigure “a” because of the creation of defects in the ZnO. The presence of optical absorption edge Fe-doped nanocrystals shows that grown nanocrystals are proper for photocatalytic performance in both U.V. and visible regions. A 3d transition in O (2p) → Zn from V.B. to C.B. creates a maximum peak absorption. The characteristic d-d transitions in high spin states of Fe^2+^ in the tetrahedral oxygen coordination are thought to be the cause of the green color of Fe-doped ZnO nanoparticles. The observed changes in the nanoparticle absorption spectra indicate that Fe^2+^ ions successfully replaced Zn^2+^ inside the hexagonal ZnO wurtzite structure. The energy bandgap E_g_ of the Fe-doped ZnO nanoparticles was computed based on Tauc’s relation. Figure S1a,b exhibited the presence of Fe around 800 eV, as shown in b.s.

### 3.4. DFT Calculations

Zinc oxide is a wide-bandgap semiconductor material with a direct bandgap of experimentally about 3.4 eV. It has a high breakdown electric field strength and a large exciton binding energy, which makes it suitable for optoelectronic devices. In terms of its electronic structure, ZnO has a hexagonal wurtzite crystal structure with a lattice constant of about 3.25 Å. However, the first-principles calculations within different approximations have been successfully applied to study band gap, which estimates the band gap from 0.68 to 1.15 even with DFT–LDA+U approach [14,15,16]. Herein, we compute the band structure of wurtzite ZnO based on different approximations of GGA/PBE, GGA/PBESOL, m-GGA/RSCAN, and Hybrid/HSE06, which predict direct band gap of 0.78, 0.68, 1.20, and 1.98, respectively. For the first time, the non-local exchange approximation of hybrid is strongly correlated with the experimental values for ZnO, and it can significantly improve the calculation of strong electron-electron correlation transition metal localization. 

**Figure 5:**
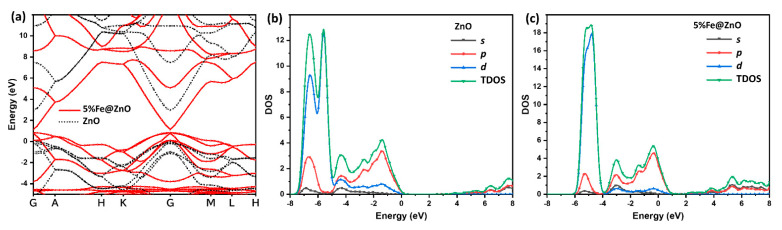
Calculated band structure of wurtzite ZnO and 5%Fe doped ZnO (**a**); Total and partial density of state of ZnO without Fe (**b**); and with 5% Fe (**c**).

As presented in Figure 5a, the bandgap of ZnO is located at the zone center of the Brillouin zone, and the valence band maximum is located at the zone edge. The conduction band minimum is located at the Γ point, which is the center of the Brillouin zone. In addition, doping zinc oxide (ZnO) with 5% iron (Fe) can modify its electronic structure and optoelectronic properties. Iron-doped ZnO (Fe@ZnO) is a p-type semiconductor, which means that it has an excess of holes (positive charge carriers) in the valence band. The band structure of 5% Fe@ZnO is similar to that of undoped ZnO, with a direct bandgap at the zone center of the Brillouin zone and a valence band maximum at the zone edge. However, the addition of Fe ions as dopants introduces additional energy levels in the bandgap, which can shift the position of the valence and conduction bands relative to the Fermi level. This can affect the electrical conductivity and optical absorption of the material, which is in good agreement with optical results and photodegrdation application.

**Figure 6:**
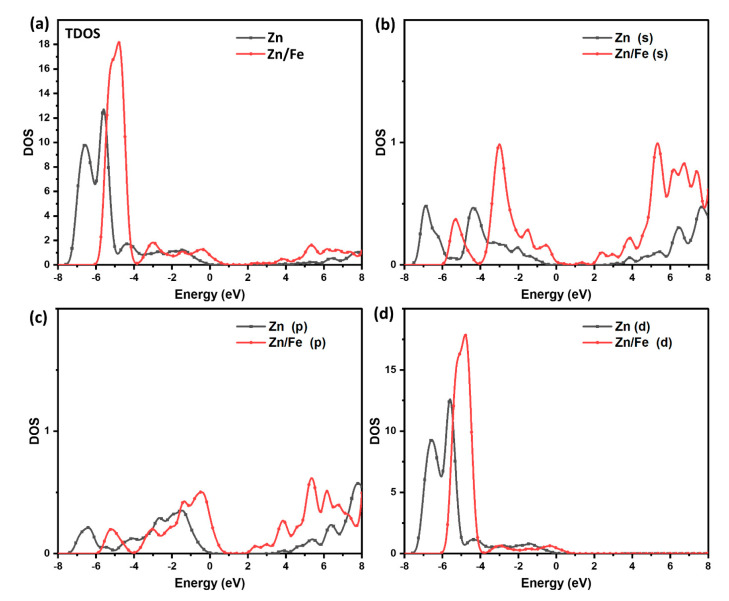
Calculated TDOS and PDOS of ZnO in comparison with 5%Fe doped ZnO, with TDOS-Zn (**a**); s-orbital contributions for Zn atom and doped 5%Fe@Zn (Zn/Fe) (**b**); p-orbital contributions (**c**); and d-orbital contributions (**d**).

**Figure 7:**
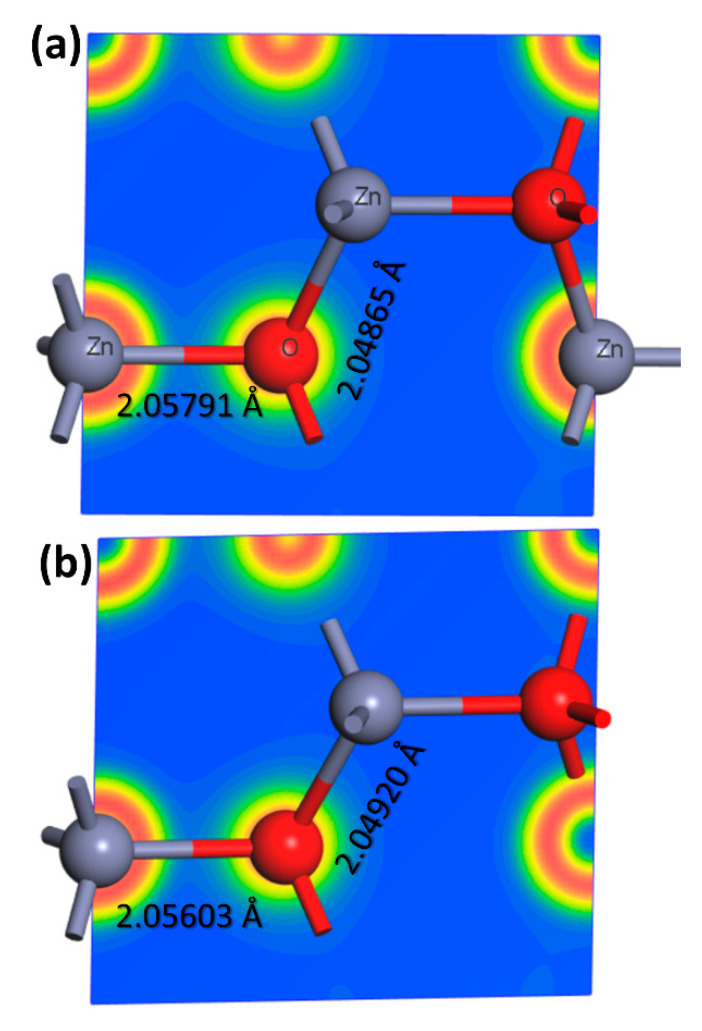
Charge density distribution at the same plan for both ZnO (**a**); and 5%Fe doped ZnO (**b**).

The density of states (DOS) of ZnO is characterized by a large valence band with mainly O 2p orbitals and a small conduction band with mainly Zn 4s and 4p orbitals. The valence band is separated from the conduction band by a wide bandgap, which makes ZnO a wide-bandgap semiconductor. The DOS of ZnO is relatively flat in the valence band, but it increases sharply near the bandgap. This leads to a high density of states at the band edge, which is known as the “Sawtooth” shape of the DOS. However, the DOS of Fe@ZnO is also modified by the presence of Fe ions. The dopants introduce additional energy levels in the bandgap, which can increase the overall density of states in the material. The DOS of Fe@ZnO is characterized by a large valence band with mainly O 2p and Fe 3d orbitals and a small conduction band with mainly Zn 4s and 4p orbitals, as shown in Figure 6. Further, ZnO and Fe@ZnO structures were relaxed to determine the charge distribution. The charge density distributions for both configurations were calculated along the same plane, as shown in Figure 7. The obtained data show a slight displacement of the Zn/Fe locations, as well as an expansion of the Zn-O bond lengths due to the doping iron, which is in a good agreement with XRD analysis.

In addition, the DFT calculations of optimized structures for both doped and undoped-ZnO show the lattice parameter “a” decreasing from 3.370135 Å to 3.360115 Å, respectively. This is in contrast to an increase in lattice parameter “c” from 5.398043 Å to c = 5.43230 Å for ZnO and Fe@ZnO, respectively. The results reported here provide further evidence for XRD Rietveld analysis. 

### 3.5. Photocatalytic Performance

**Figure 8:**
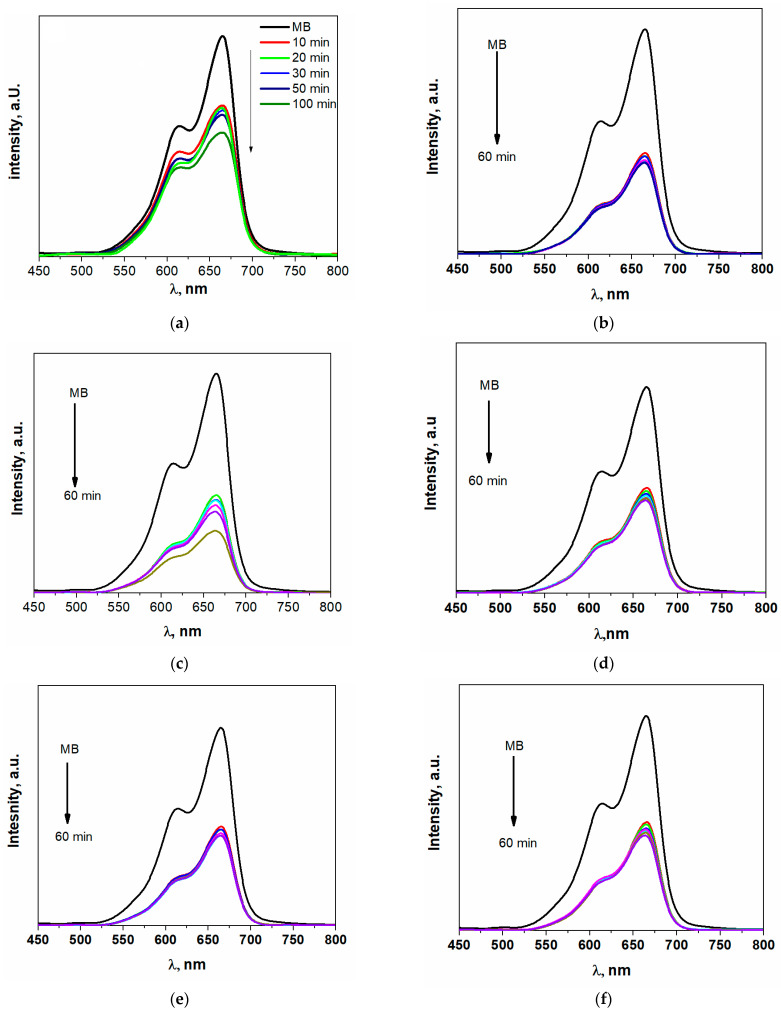
The photodegradation of M.B. by different concentrations of Fe in Zn_1−x_Fe_x_O (**a**) x = 0, (**b**) x = 0.03, (**c**) x = 0.05, (**d**) x = 0.07, (**e**) x = 0.1 and (**f**) x = 0.15.

In addition, the photocatalytic activity of ZnO was assessed by varying the number of additional Fe added under the optimal prior M.B. removal conditions. U.V.–vis absorption spectra of M.B. before and after Zn_1−x_Fe_x_O stirring were explained by the findings in Figure 8a–f. With increasing exposure time, M.B. displayed a maximum absorption band of 665 nm, which decreased in intensity. The photocatalytic performance of Zn_0_._95_Fe_0.05_O composites is superior to that of pure ZnO. The enhancement in the photocatalytic performance of ZnO is due to doped with 5% Fe since the energy gap of ZnO was decreased to 2.9 eV. The photocatalytic performance of a photocatalyst strongly depends on its electronic band structure and bandgap energy, E_g_. For an efficient photocatalyst, the bandgap energy should be smaller than 3 eV to extend the light absorption into the visible region to utilize solar energy efficiently. As exhibited in Figure S2, the increase in the Fe concentrations demonstrated a significant decrease in the intensity PL, suggesting a larger barrier to charge recombination.

**Figure 9:**
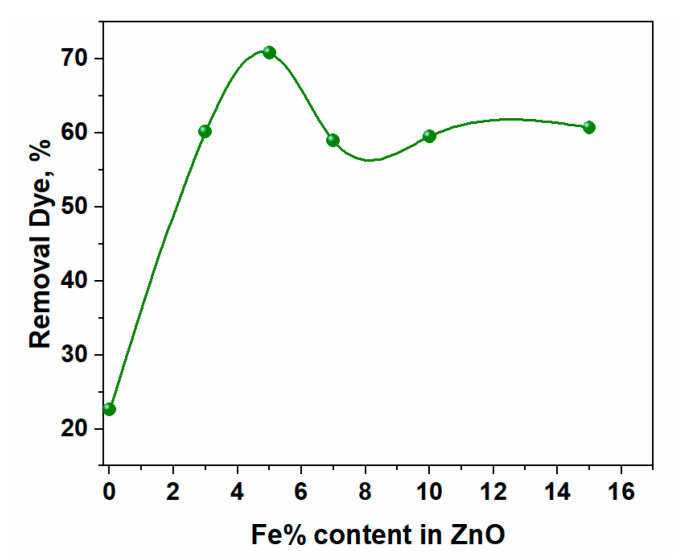
The efficiency of different concentrations of Fe in ZnO.

**Figure 10:**
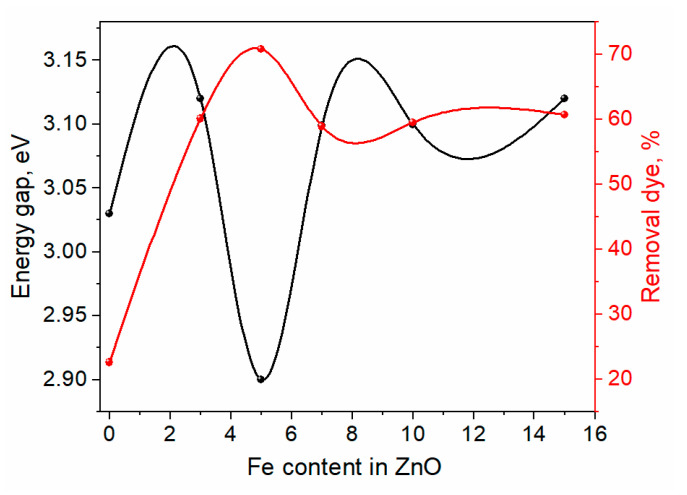
The relation between energy gap and removal dye efficiency as a function of Fe concentrations in ZnO.

Figure 9 showed that ZnO MB-degradation effectiveness was around 44.5%. When ZnO doped Fe was lower, the photocatalytic performance was strengthened with an increase in the Fe concentrations, where the MB-degradation efficiency was raised to around 72% in the instance of Zn_0.95_Fe_0.05_O as shown in Figure 8c due to the energy gap of this composition at approximately 2.9 eV, as seen in Figure 10. However, the increase in Fe content led to a rise in the energy gap over 3 eV, and the photocatalytic activity decreased with increased Fe content. As a result, it is probable that the high concentrations of Fe in a composite may increase the likelihood of recombination of photo-generated electron-hole pairs in the composite.

As can be seen in Figure 9, the initial M.B. concentration increases as the M.B. decolorization percentage decreases. Because the photons-path length through the MB-solution was shortened and the active surface sites of ZnO doped Fe were covered, this behavior is acceptable. This process reduces the amount of ultraviolet radiation absorbed by Zn_1−x_Fe_x_O particles, which in turn reduces the creation of electron-hole pairs (i.e., reactive radicals), lowering the amount of M.B. photodegradation [23]. This means that MB-dye solution with a Zn_1−x_Fe_x_O photocatalyst should have a concentration of 10 ppm or less. Thus, the best concentration is dropping by 5% of Fe. 

## 4. Conclusions

The pristine ZnO and Fe-doped ZnO were prepared by rapid sol-gel combustion method at one hour. The XRD confirmed that all materials have a single hexagonal phase without any other impurities by other crystalline phases. The particle size was increased by increasing the amount of Fe doping, but the strain was decreased by increasing the quantity of Fe. The photo of TEM confirmed that the doping of Fe led to an increase in the size of ZnO. The absorption band of ZnO was decreased by increasing the amount of Fe doping, and the energy gap changed from 3.12 to 2.9 eV. The pure ZnO exhibited poor efficiency in removing the M.B. dye since the photocatalysis efficiency was 45%. However, the increase in the quantity of Fe led to enhance the photocatalysis efficiency of ZnO by up to 5% since the efficiency was reached ~72. However, the increase in the amount of Fe by over 5% led to decreased efficiency.

## Data Availability

The data will be available under reasonable requirements.

## Supplementary Materials

The following supporting information can be downloaded at: https://www.mdpi.com/article/10.3390/ma16072676/s1, Figure S1: (a,b) exhibited the presence of Fe around 800 eV, as shown in b; Figure S2: PL spectra of Zn_1−x_FexO.
