# Supplementary material for: Optical Band Gap Tuning, DFT Understandings, and Photocatalysis Performance of ZnO Nanoparticle-Doped Fe Compounds
Source: Materials (Basel). 2023 Mar 28;16(7):2676. doi: 10.3390/ma16072676 (PMC10096406; doi:10.3390/ma16072676)
Supplement: Supplementary file 1 [file materials-16-02676-s001.zip › materials-2190648-supplementary.pdf]

Figure S1(a,b) exhibited the presence of Fe around 800 eV, as shown in b.

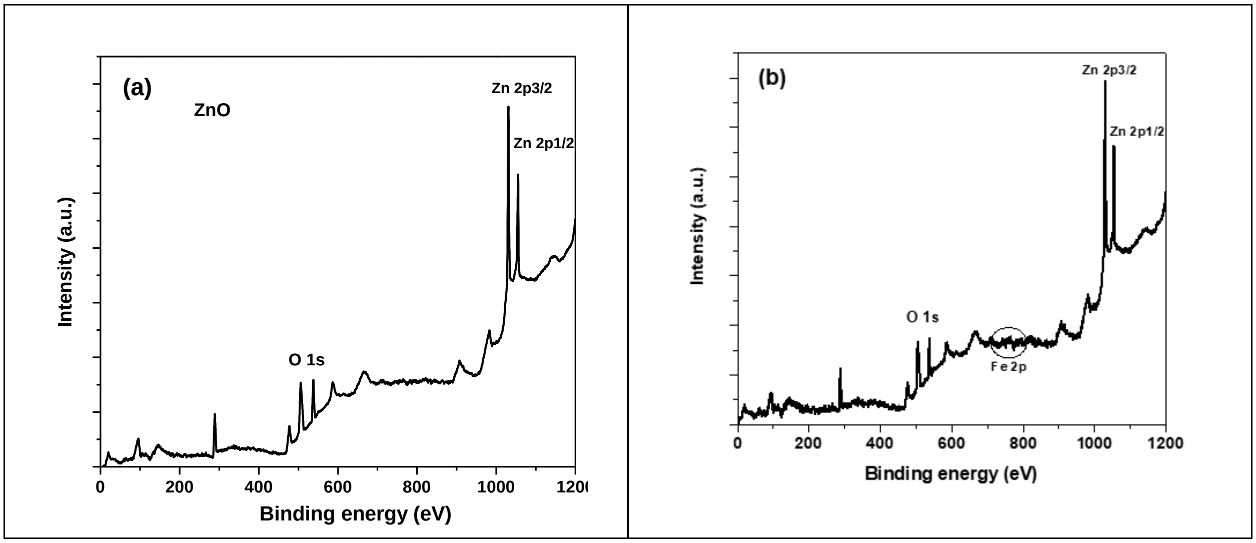

As exhibited in figure S2, the increase in the Fe concentrations demonstrated a significatint decreas in the intensity PL, suggesting a larger barrier to charge recombination.

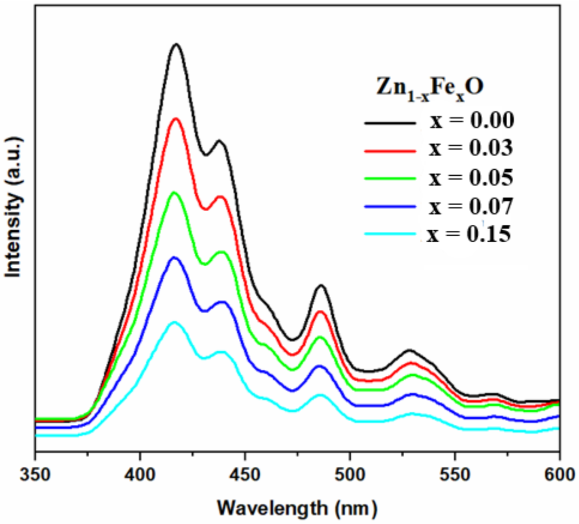

Figure S2: PL spectra of $Zn_{1-x}Fe_xO$
